# Synthetic directed evolution for targeted engineering of plant traits

**DOI:** 10.3389/fpls.2024.1449579

**Published:** 2024-09-02

**Authors:** Ahad Moussa Kababji, Haroon Butt, Magdy Mahfouz

**Affiliations:** Laboratory for Genome Engineering and Synthetic Biology, Division of Biological Sciences, 4700 King Abdullah University of Science and Technology (KAUST), Thuwal, Saudi Arabia

**Keywords:** CRISPR-Cas9, synthetic directed evolution, CRISPR-directed evolution, rational protein design, trait engineering, crop breeding, noncoding DNA, regulatory elements

## Abstract

Improving crop traits requires genetic diversity, which allows breeders to select advantageous alleles of key genes. In species or loci that lack sufficient genetic diversity, synthetic directed evolution (SDE) can supplement natural variation, thus expanding the possibilities for trait engineering. In this review, we explore recent advances and applications of SDE for crop improvement, highlighting potential targets (coding sequences and *cis*-regulatory elements) and computational tools to enhance crop resilience and performance across diverse environments. Recent advancements in SDE approaches have streamlined the generation of variants and the selection processes; by leveraging these advanced technologies and principles, we can minimize concerns about host fitness and unintended effects, thus opening promising avenues for effectively enhancing crop traits.

## 1 Introduction

**Figure 1:**
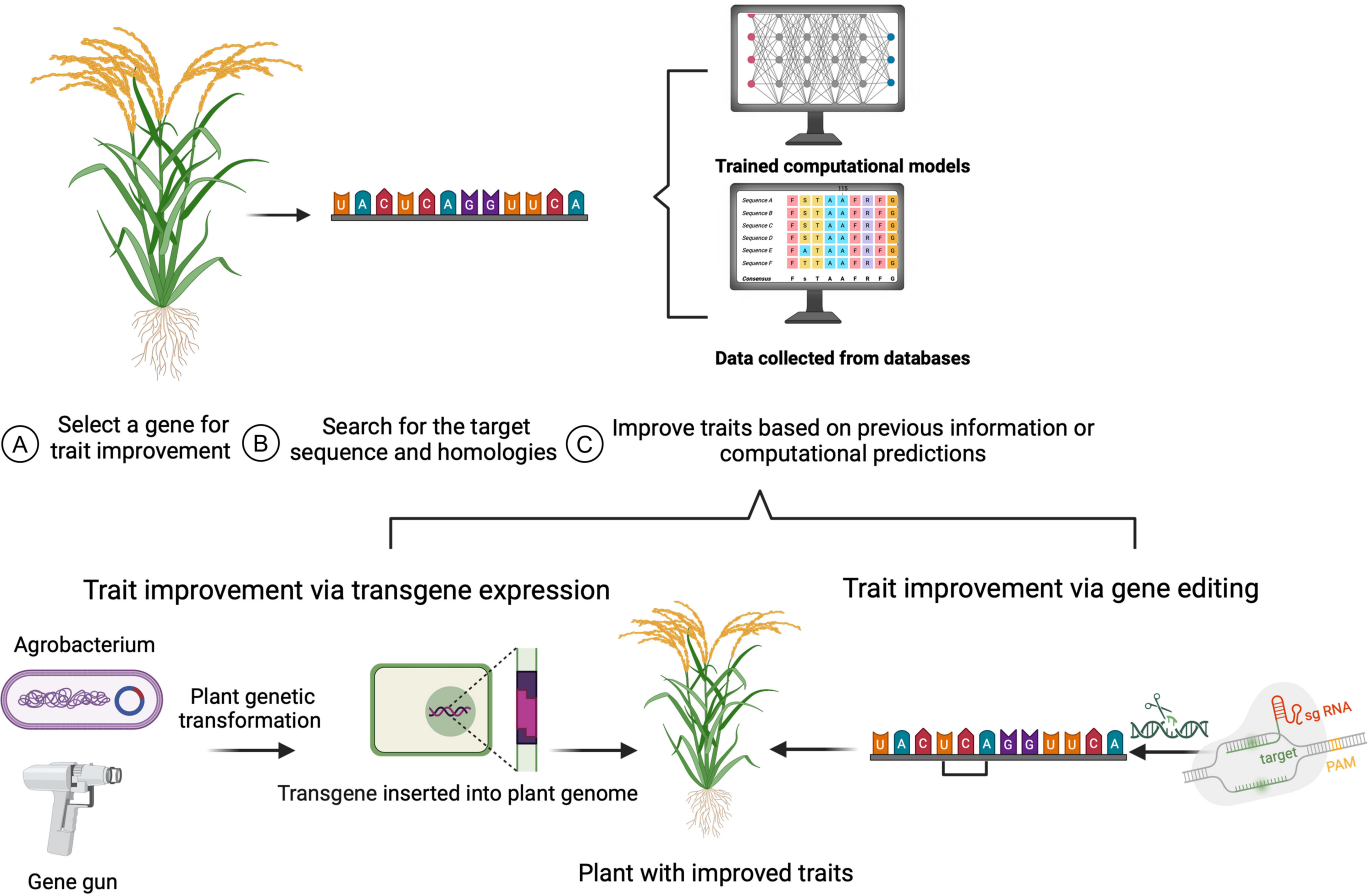
Target gene selection for plant trait engineering. **(A)** A candidate gene is selected with known activity related to a desired agronomic trait. **(B)** The potential target sites within the gene are identified, and **(C)** analyzed based on prior studies and/ or computational prediction tools. A transgene can be introduced to the plant genome via plant transformation methods, such as particle bombardment using a gene gun or Agrobacterium-mediated transformation (bottom left). Gene editing tools like CRISPR/Cas9 system, are used for genome modification (bottom right). The crop plants with the desired gene variants will have improved traits resulting from transgene expression or gene editing.

Plant breeding introduces genetic diversity into breeding populations by intercrossing plants with outstanding traits and then selecting progeny with desired characteristics (Zsogon et al., 2022). However, many species and loci lack sufficient genetic diversity, meaning that breeders do not have germplasm with alleles that will produce the desired characteristics. Genome engineering technologies, including the CRISPR/Cas9 system, offer new avenues to improve crop traits (Das et al., 2022) (**Figure 1**). Although CRISPR/Cas systems have been primarily used to generate loss-of-function alleles, they can also be used as a powerful tool for generating genetic diversity at specific loci. This enables new, innovative approaches such as synthetic directed evolution (SDE).

SDE uses iterative cycles of artificial diversification of a gene sequence, followed by selection for a trait of interest (*in vitro* or *in vivo*) to produce desirable alleles. This versatile approach can generate genetic diversity and drive the evolution of organisms towards desired traits (Simon et al., 2019). Recent advancements have automated all essential stages of SDE, significantly reducing the time required for variant generation and minimizing the effort demanded from researchers during the transfection, diversification, and screening stages (Packer and Liu, 2015). Modern SDE methods do not require prior knowledge of the gene background or structure as necessary for rational engineering. To generate variants, when specific genes are targeted via these modern SDE techniques, there are fewer concerns regarding host fitness and viability. Instead, variants are selected based on their performance under a range of selective pressures. However, SDE also presents some challenges, including designing selective assays, generating large, unbiased DNA libraries, avoiding false signals, and significant manual effort during mutagenesis and selection (Chen, 2001).

Here, we focus on SDE approaches for protein engineering to generate crop lines with improved agronomic and climate-resilient traits. We shed light on current efforts to harness SDE and expand genetic diversity for crop improvement. The SDE technologies have been utilized in plants to engineer and improve herbicide resistance. Later, we provide an overview of engineering *cis*-regulatory elements (CRE) and motifs in plant trait improvement. We discuss the computational tools and databases for predicting *cis*-regulatory elements in plants. At the end, we discuss with suitable examples how targeting promoter regions, upstream-open reading frames (uORF), enhancers, transcription factors, 5´-UTR, and 3´-UTR can improve plant traits. We discuss the constraints and prospects inherent in directed evolution for trait improvement in crops.

## 2 Synthetic directed evolution

**Figure 2:**
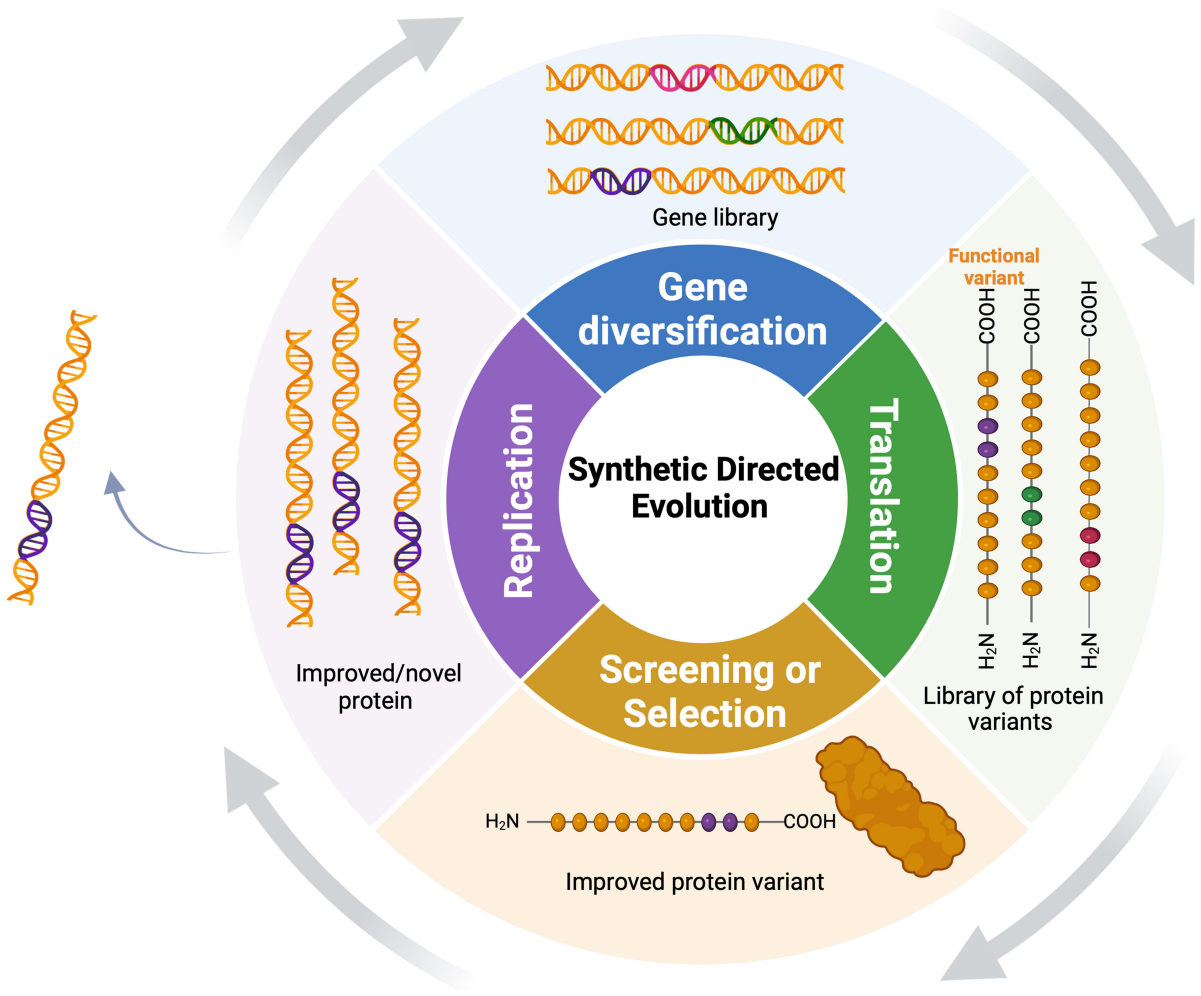
Synthetic directed evolution (SDE) for protein engineering: The target gene undergoes diversification to yield a pool of gene variants, followed by iterative selection to enrich for variants with enhanced functional qualities under selection. Functional variants undergo continuous diversification, exponentially amplifying their superior characteristics.

SDE has two basic steps: in the diversification stage, researchers introduce changes in specific gene sequences, generating a library of gene variants encoding a diverse set of protein variants (**Figure 2**). The diversification step can involve mutagenesis at various levels, including single genes, multiple genes, genomes, or within complex pathways, employing direct (gene-focused) or random (whole-genome) platforms (Wang et al., 2021). Depending on the methods used for diversification, these variants can be handled *in vitro*, transformed into cells or organisms, or generated *in vivo*. In the selection stage, the host population is subjected to a specific selection pressure to identify individuals with the desired traits (Simon et al., 2019). Selection and screening enable the isolation and recovery of only those variants that exhibit the desired functional characteristics. Subsequently, selected variants undergo multiple rounds of SDE to further enrich and amplify desired traits.

As the field of SDE has advanced, there has been a transition from manipulating individual components and modifying products to address more complex pathways. This transition was highlighted by the awarding of the Nobel Prize in Chemistry to Frances H. Arnold, George P. Smith, and Sir Gregory P. Winter in 2018, for their work on development of new pharmaceutical enzymes through directed evolution (Arnold, 2019; Service, 2018).

Although SDE has shown great promise in bacteria, yeast, and mammalian cell lines, it has fewer success stories in plants and most of these have been proof-of-concept studies generating herbicide tolerance by mutating genes known to confer this phenotype. Nevertheless, leveraging SDE has the potential to enable the discovery of novel traits, broadening the spectrum of useful traits, and accelerating trait development in targeted crops essential for food security (Rao et al., 2021; Butt et al., 2020; Garcia-Garcia et al., 2022; Wright et al., 2023). Moreover, the integration of SDE principles with biotechnology methodologies has revealed extensive potential for generating protein variants, enhancing natural enzyme catalytic activity, probing desired phenotypes, and introducing novel traits in diverse living organisms.

### 2.1 Methods and strategies for synthetic directed evolution

Diversification can be performed with various strategies, including random mutagenesis, DNA recombination and CRISPR/Cas-based methods (Wang et al., 2021). The groundwork for SDE was laid in the early 1970s when a chemical mutagens like ethyl methanesulfonate (EMS) or l-methyl-3-nitro-l-nitrosoguanidine (NG) induced a new functional phenotype in *Aerobacter aerogenes* (Lerner et al., 1964). Use of a phage-expressing library as an *in vitro* diversification method yielded novel and desirable properties for various targeted genes (Levisohn and Spiegelman, 1969). Subsequent *in vitro* studies introduced mutations into specific genetic regions, followed by iterations of mutation and *in vivo* screening. This led to the gradual accumulation of advantageous mutations in a strategy termed combinatorial cassette mutagenesis (Reidhaar-Olson and Sauer, 1998; Mills et al., 1967).

Error-prone polymerase chain reaction (PCR) has emerged as a groundbreaking technique for *in vitro* random mutagenesis and diversifying sequences across multiple rounds, either segmentally or entirely. CasPER (Cas9-mediated protein evolution reaction) employ CRISPR/Cas9 for DSB at genomic targets to integrate mutagenized 300–600 bp linear DNA donors for the directed evolution of enzymes in native genomic contexts. The CasPER technique used error-prone PCR to generate combinatorial libraries serving as templates for introducing diversity into two essential enzymes within the mevalonate pathway (Jakociunas et al., 2018). However, random mutagenesis using error-prone PCR has limitations such as the low mutation rate, restricted amino acid variants along the target gene, and the low probability of encountering adjacent mutations (Chen and Arnold, 1993, 1991; Wen et al., 2007). One variation of epPCR is site saturation mutagenesis (SSM) which displayed substantial enhancement in functional performance. OmniChange, a multi-site-saturation method, uses PCR and synthetic DNA oligonucleotides for simultaneous and efficient saturation of five independent codons (Dennig et al., 2011).

In the mid-1990s, the recombination approach exemplified by DNA shuffling was established as an *in vitro* diversification strategy, exchanging larger fragments from similar genes to generate a library of functional variants (Stemmer, 1994a, b; Crameri et al., 1998; Jermutus, 2000). SDE through genome shuffling or inter-strain fragment shuffling has been widely employed in various bacterial strains and other organisms (Zhang et al., 2002; Patnaik et al., 2002; Schmidt-Dannert et al., 2000; Kumamaru et al., 1998; Biot-Pelletier and Martin, 2014). The merging of enhanced variations via DNA recombination after selection enables the integration of functional and improved variants while removing harmful ones, resulting in significant trait improvements over the course of multiple generations.

Another approach uses oligo libraries suitable for recombineering and CRISPR-based genome editing. It generates unique collections of codons for each amino acid to be mutated, ensuring the desired level of redundancy necessary to efficiently achieve a diverse population. Excluding the wild-type amino acid helps reduce library size without compromising diversity. As an application, λ-red recombineering via CRISPR-based-selection was accomplished by creating an engineered cell library devoid of wild-type amino acids or stop codons, lacking redundant codons, and incorporating highly utilized codons in *E. coli* (Pines et al., 2015).

The Multiplex Automated Genome Engineering system (MAGE) was employed to create rapid continuous diversification and mutagenesis of specific sites within the bacterial genome, generating variants with combinatorial libraries *in vivo* (Wang et al., 2009). Because MAGE can introduce short oligonucleotides to create a library of the desired genomes only in *E. coli*, Co-Selection MAGE (CoS-MAGE) was modified to include targeting regulatory elements of the desired targeted gene (Wang et al., 2012). Similarly, the Directed Evolution with Random Genomic Mutations technique (DIvERGE) allows for the evolution of multiple loci in their native genomic context within a targeted coding gene and its promoter regions (Nyerges et al., 2018).

Exploring combinatorial sequence space experimentally can be resource-intensive. To overcome this issue, machine learning models were initially trained on tested variants to swiftly generate computational libraries. For example, a computational model was trained to predict the best combinatorial library for the human GB1 binding protein based on a sequence-function dataset, followed by using the predicted library in the evolution round for validation. As a proof of concept, an enzyme for stereodivergent carbon–silicon bond formation, a new-to-nature chemical transformation, was evolved to produce a pair of enantiomers. This method accurately predicted libraries with enriched enzyme activity, leading to the identification of variants with selective catalytic properties (Wu et al., 2019). A basic machine learning protocol was extensively reviewed and was supported by case studies in which models can guide protein engineering and enhance SDE in various species (Yang et al., 2019).

CRISPR/Cas-based techniques have revolutionized the field of SDE. Designed with precision, the CRISPR/Cas9 system not only can induce both random and localized diversification but also can serve as an effective guiding mechanism for other diversification agents like base editors and EvolvR (Doudna and Jiang, 2017). A notable example is the successful editing of gram-negative bacteria *Pseudomonas aeruginosa* using the methods pnCasPA-BEC and pCasPA/pACRISPR. In pnCasPA-BEC system, a cytidine deaminase APOBEC1 is fused with Cas9-nickase that enables highly efficient gene inactivation and point mutations in a variety of Pseudomonas species. The pCasPA/pACRISPR system harnessed the CRISPR/Cas9 and the phage λ-Red recombination systems to accelerate a wide variety of investigations, such as bacterial physiology study, drug target exploration, and metabolic engineering (Chen et al., 2018). The EvolvR technology uses nCas9 (D10A) along with a bacterial error-prone DNA polymerase (PolI3M) and a thioredoxin-binding domain (TBD), in addition to a synthetically designed sgRNA, allowing ongoing localized sequence diversification within bacterial targets (Halperin et al., 2018). However, the editing window was limited to −13 to −18 bases upstream of the protospacer adjacent motif (PAM) sequence.

Producing a diversified gene sequence is only the first step; for SDE, the phenotypes resulting from each modification must undergo selection to identify variants that produce phenotypes of interest. Phage-assisted continuous evolution (PACE) is an M13 phage-based technique for the continuous directed evolution of proteins. PACE revealed various advantageous and applications in diversifying peptides, including enzymes, antibodies, and receptors, through iterative phage propagation and selection cycles. PACE has the capacity to evolve any protein associated with the expression of an essential phage gene (Popa et al., 2020; Miller et al., 2020). A recent comparison between Phage-Assisted Continuous Evolution (PACE) and Phage-Assisted Non-Continuous Evolution (PANCE) revealed various advantages and applications in diversifying the selection cycles. PACE induces random mutations within the DNA sequence in a bioreactor, generating new variants at a much higher rate than natural evolution. The expressed proteins are then selected based on their fitness *in situ*. In contrast, the PANCE system allows for multiplexing of phage-based evolution and can enhance the evolution of desired targets with initially low starting activity. Hence, PANCE is slower and is more time-intensive compared to the PACE system (Popa et al., 2020; Miller et al., 2020).

To illustrate the diverse, complex approaches used for SDE in eukaryotes, we have described some selected examples in the following section.

### 2.2 Examples of synthetic directed evolution in eukaryotes

Among eukaryotes, a major success of SDE has been reported in yeast cells. The yeast OrthoRep system utilizes a highly error-prone orthogonal DNA polymerase (TP-DNAP1) for *in vivo* diversification of desired DNA segments independent of the remaining host genome sequence. These genes are located on orthogonal, non-genomic plasmids that replicate using error-prone DNA polymerases (Ravikumar et al., 2018). As a proof of principle, the OrthoRep system evolved primary metabolic enzymes, including thiazole synthase (THI4), aiming to modify plant enzymes for plant applications, and adapt enzymes from prokaryotes to function effectively in mild, plant-like conditions (Garcia-Garcia et al., 2022). A novel orthogonal replication system, TP-DNAP1^Y427A^, was developed as a platform for *in vivo* continuous evolution in *Saccharomyces cerevisiae*. This system allows for independent manipulation of replicative properties compared to the host organism. The mutation rate per base of TP-DNAP1^Y427A^ is approximately 400-fold higher compared to the host genome (Ravikumar et al., 2014).

TRIDENT (TaRgeted *In vivo* Diversification ENabled by T7 RNAP) is another platform for continuous diversification of target genes at mutation rates one-million fold higher than natural genomic error rates. The TRIDENT system is designed to enable *in vivo* diversification by integrating an error-prone base editor with a DNA-dependent RNA polymerase derived from a bacteriophage (T7RNA polymerase) (Cravens et al., 2021). This fusion is under the control of a T7 phage promoter that is specifically inserted upstream of the desired target gene. The TRIDENT system introduces substitutions along the template strand during transcription, without creating double-strand breaks (DSBs). In this system, T7-RNA polymerase is fused to pmCDA1-CBE along with repair factors, synthetic inducible promoters, and a distributed UNG strain (*ungΔ* strain). Uracil DNA Glycosylase (UNG) encodes the primary enzyme responsible for DNA repair of uracil and disruption of the UNG1 gene improves C/G mutagenesis (Kurt et al., 2021). Mutational diversity is tunable within this system due to DNA repair factors that localize to the sites of deaminase targeting in the yeast cell. Using the TRIDENT system in yeast produced A:T and C:G mutations at similar rates across multiple kb of DNA fragments (Cravens et al., 2021).

SDE has also been developed and successfully employed in mammalian cell lines. The T7 RNA polymerase-driven Continuous Editing (TRACE) system has been harnessed for continuous mutagenesis applications. TRACE uses a T7 polymerase fusion with two naturally occurring cytosine deaminases, catalytic polypeptide (APOBEC1) and a hyperactive mutant of activation-induced cytidine deaminase (AID*Δ), which are independently used for continuous mutagenesis. The base editing window downstream of the T7 promoter reached 2 kb of the targeted DNA, and continuous nucleotide diversification throughout 20 cell generations was seen (Chen et al., 2020).

An example of a CRISPR-based screening tool is the Targeted AID-mediated Mutagenesis (TAM) system, which provides a genetic tool to generate a broad spectrum of diverse variants suitable for gain-of-function screening. Unlike other large-scale screening methods, which mainly rely on loss-of-gene function or changes in gene expression, the TAM tool uses a fusion of AID-BE with catalytically inactive dCas9 for high-throughput screening of functional variants. This led to the identification of known and novel variants conferring imatinib resistance in chronic myeloid leukemia cells (Ma et al., 2016). Unlike TAM where AID is fused with dCas9, a modified approach CRISPR-X was used, where a hyperactive AID fused to MS2 binding protein recruited through sgRNA containing MS2 hairpins. The dCas9 variant complexes with the sgRNA and guides the sgRNA-MS2-AID complex to the desired genomic location (Hess et al., 2016). The CRISPR-X extended editing window responding to the PAM sequence compared to TAM. Repurposing the somatic hypermutation machinery, CRISPR-X facilitates *in situ* protein engineering, generating diverse libraries at localized points through multiplexing or multiple rounds of sgRNA library induction. It can target multiple genomic locations simultaneously, with minimal off-target effects. By utilizing the CRISPR-X system, researchers successfully evolved the cancer therapeutic bortezomib by focusing on *PSMB5*, identifying both known and novel mutations conferring bortezomib resistance (Hess et al., 2016).

The recently developed CRISPRres (CRISPR-induced resistance) system facilitates swift diversification and identification of drug resistance mutations in vital genes. CRISPRres creates genetic variation via CRISPR-Cas-induced non-homologous end-joining (NHEJ) repair to generate a wide variety of functional in-frame mutations. One of the applications of CRISPRres is to target the anticancer agent *KPT-9274*, pinpointing nicotinamide phosphoribosyltransferase (*NAMPT*) as its primary target. Using tiling libraries of sgRNAs directed at multiple genes that conferred resistance to the anticancer drug bortezomib, CRISPRres produced multiple in-frame mutations within the *PSMB5* coding sequence, a key focal point in resistant-treated cells (Neggers et al., 2018).

Over the last decades, viruses have played pivotal roles in gene therapy and vaccine development within the emerging field of synthetic virology. Enhanced diversification by the naturally error-prone Sindbis viral RNA-dependent RNA polymerase, known as viral evolution of genetically actuating sequences (VEGAS), enables straightforward directed evolution in mammalian cells. VEGAS has been successfully applied to evolve TFs (English et al., 2019). VEGAS evolves within the signaling framework of the host cell, wholly dependent on the host cell, and selection is constant and highly mutagenic, enabling it to overcome many of the pitfalls inherent to complex fitness landscapes.

### 2.3 Synthetic directed evolution in plants: herbicide tolerance

Because of the ease of selection, many proof-of-concept studies in plants have used SDE to induce herbicide tolerance. Moreover, most of the plant genes tested were evolved in bacteria and later introduced into plant cells (Hendel and Shoulders, 2021; Rodrigues et al., 2021). The sequences evolved in bacterial systems were often unstable in plant cells due to the complex nature of internal pathways, codon usage patterns, RNA instability, as well as the unique anatomy and physiology of plants that ultimately hindered the broader adoption of the SDE tools to plant models. Devising technologies for high-efficiency gene sequence variation within plant cells coupled with selection pressure would enable the synthetic-directed evolution for trait discovery and engineering in plants. Developing, testing, optimizing, and establishing technologies for high-efficiency localized sequence variation coupled with selection pressure are indispensable to evolve gene variants for traits of value.

**Figure 3:**
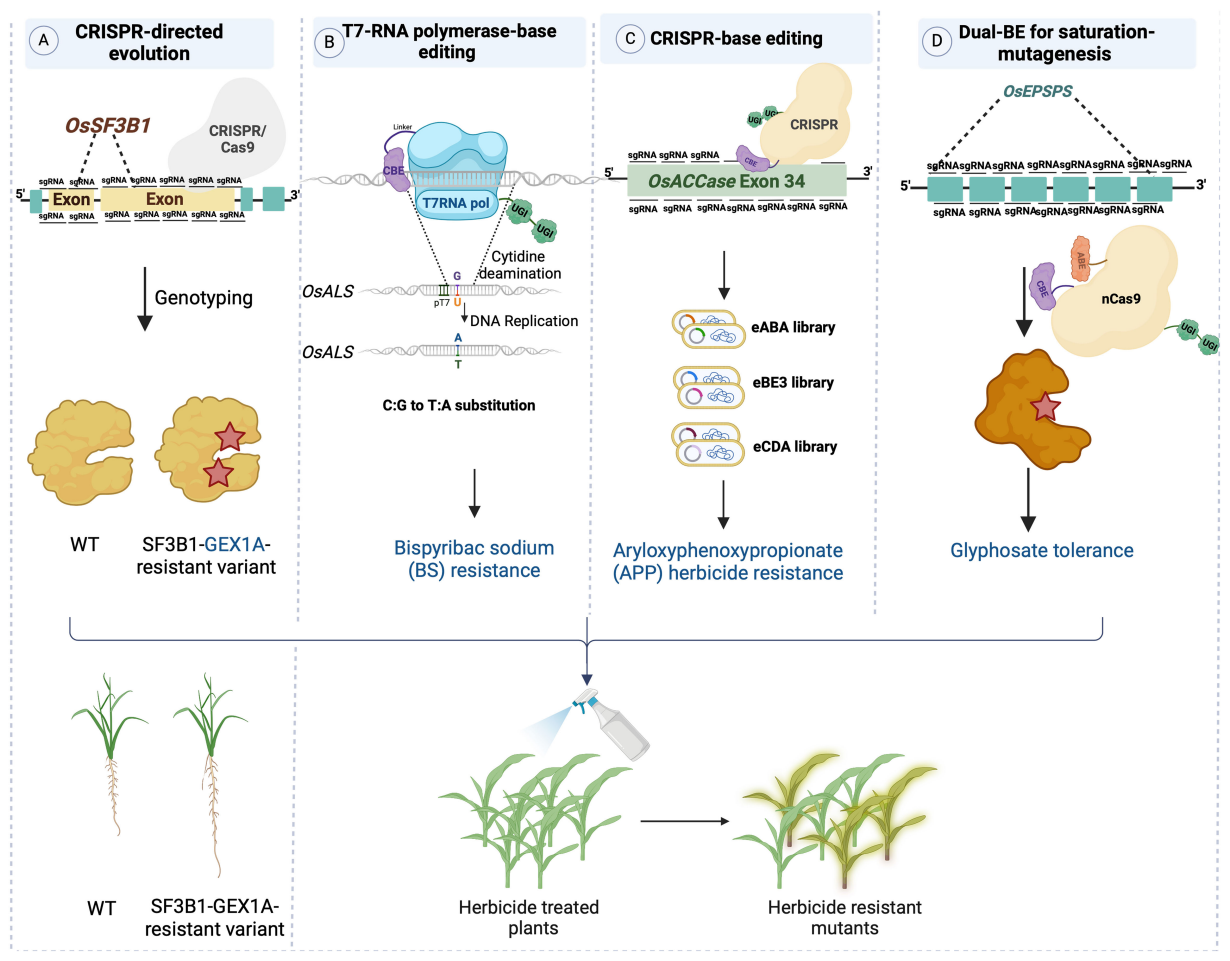
Platforms for directed evolution of plant traits platforms for directed evolution of plant traits. **(A)** CDE: a targeted library of single guide RNAs (sgRNAs) was transformed into Agrobacterium tumefaciens for editing OsSF3B1 in rice. To accelerate evolution, plants were regenerated under selective pressure from the splicing inhibitor herboxidiene (GEX1A), resulting in the recovery of protein variants that conferred tolerance to GEX1A. **(B)** T7pol-CBE: a chimeric protein consisting of a cytidine deaminase and T7 RNA polymerase was harnessed for targeted mutagenesis of the OsALS coding sequence under control of the T7 promoter. This system generated OsALS variants that confer resistance to the herbicide Bispyribac sodium (BS). **(C)** CRISPR-BE: a sgRNA library was introduced as a pool along with CBE (cytosine base editor), which targeted OsACC exon 34. The resulting rice lines were rendered resistant to ACCase-inhibiting chemicals such as the herbicide aryloxyphenoxypropionate herbicide. **(D)** Dual-BE: a OsEPSPS coding sequence was targeted via STCBE‐2 system, consisting of Cas9-nickase and dual base editors (CBE and ABE) with dual UGI fragments. A tailed library focusing on glyphosate‐binding domains induces C:T and A:G base substitutions, resulting in glyphosate-resistant rice plants.

SDE was initially established by targeting a splicing factor called *SF3B1* in rice using CRISPR/Cas9 system (Butt et al., 2019; Zhang and Qi, 2019) (**Figure 3A**). *SF3B1* is a subunit of the SF3B spliceosome within the U2 small nuclear ribonucleoprotein (U2SnRNP) complex. A library of sgRNAs was employed to target *SF3B1* in rice. The edited sequences were generated randomly through NHEJ repair pathway. This CDE (CRISPR-directed Evolution) platform used to produce SF3B1-mutant variants showing varying levels of resistance to the splicing inhibitor herboxidiene (*GEX1A*) (**Table 1**) (Butt et al., 2019).

**Table 1:** Herbicide-resistant point mutations engineered in Plants.

Herbicide gene	Type of mutation	Bioengineering Tool	Herbicide	Species	Reference
*SPLICING FACTOR 3B SUBUNIT 1 (OsSF3B1)*	K1049R+K1050E+G1051H/H1048Q+A1064S	CRISPR-directed evolution	GEX1A/Pladienolide B	Rice	(Butt et al., 2019, 2021)
*PHD finger protein 5A (OsPHF5A)*	Y36C	Overexpression			
Acetolactate synthase (ALS)	A96V	Target-AID	Imazamox	Rice calli	(Shimatani et al., 2018)
	G95A	*In vitro* ALS activity assay and overexpression in rice using ubiquitin promoter	pyrimidinyl carboxy (PC): Bispyribac sodium (BS)	*Oryza sativa*	(Okuzaki et al., 2007)
	A121T P187S S652N	Site-directed mutagenesis	Londax (sulfonylurea) Cadre (imidazolinone)	*Nicotiana benthamiana*	(Chong and Choi, 2000)
	A122T A122 T/V A122V	Random mutagenesis Transgene expression	Sulfonylureas (SU): Chlorsulfuron Imazamox, bispyribac-sodium, and penoxsulam Pyrimidinylcarboxy(PCs)	*Raphanus raphanistrum(*Wild radish) Barnyardgrass (Echinochloa crusgalli) *Arabidopsis*	(Han et al., 2012) (Riar et al., 2013) (Shimizu et al., 2011)
	P171F	Base-editing mediated gene evolution (BEMGE)	Bispyribac sodium (BS)	*Oryza sativa*	(Kuang et al., 2020)
	P165 A/S/L/W	Base editing	Sulfonylureas (SU): Chlorsulfuron	*Maize*	(Li et al., 2020c)
	P171 S/A/Y/F G628E G629S Triple mutant (P171F+G628E+G629S)	Base editing (CBE)	Bispyribac sodium (BS) For Triple mutant: nicosulfuron, imazapic, pyroxsulam, BS, and flucarbazone	*Oryza sativa*	(Zhang et al., 2021)
	W548L/S627I (Double mutation) W548L/and S627I (Tow point mutations)	Overexpression of dual mutated gene Site-specific modification	Bispyribac sodium (BS) Bispyribac sodium (BS)	Oryza sativa L. cv. *Nipponbare* Oryza sativa L. cv. Kinmaze	(Kawai et al., 2007) (Endo et al., 2007)
	P197-(H/T/A/L/S/A/I/M/L/W) P197 S/A D376E	Target-site mutation PCR-RFLP assay QuikChange site-directed mutagenesis system	Sulfonylureas (SU) Imidazolinones (IMI) Imidazolinones (IMI Sulfonylureas (SU): (Tribenuron) Sulfonylureas (SU)	Kochia (*Kochia scoparia*) *Lolium rigidum* *Papaver rhoeas L.* (corn poppy) Weed Monochoria vaginalis (*syn. Pontederia vaginalis*)	(Warwick et al., 2008) (Anthimidoua et al., 2020) (Délyea et al., 2010) (Tanigaki et al., 2021)
	W574L	Target-Site Mutation	Imazamox	*Euphorbia heterophylla* L (Wild Poinsettia)	(Mendes et al., 2020)
	S653I	Transgene expression	Bispyribac sodium (BS) Pyrimidinylcarboxylates (PCs)	*Oryza sativa L. subsp. Japonica* *Arabidopsis*	(Shimizu et al., 2011)
	G654 E/D	Random mutagenesis	Imidazolinones (IMI)	*Setaria viridis*	(Laplante et al., 2009)
	S186P K416G L662P	Hybridization	Imazethapyr	Strawhull (SH) red rice	(Rajguru, 2005)
Acetyl-CoA carboxylase (ACCase)	W2125L W2125Q W2125 S/R I2139N C2186H G2194S G2194A C2186H	Base editing‐mediated targeted evolution	Haloxyfop‐R‐ methyl (Gallant)	Japonica rice (*Nipponbare*)	(Wang et al., 2022)
	W2125S C2186R I1879V	CRISPR-mediated base editing tools (eABE, eBE3, and eCDA)	Haloxyfop-R-methyl	*Oryza sativa*	(Liu et al., 2020)
	P1927F W2125C S1866F A1844P	STEME-1 STEME-NG	Haloxyfop	Rice protoplasts	(Li et al., 2020a)
	D2078G I1781L W2027C I2041N D2078G C2088R	*In Vitro* inhibition of ACCase activity	Clethodim APP and CHD	*Lolium rigidum*	(Yu et al., 2007)
5-enolpyruvoylshikimate-3-phosphate synthase *(EPSPS)*	D213N (glyphosate binding domain)	STCBE-2	Glyphosate	*Oryza sativa*	(Zhang et al., 2023)
	T102I P106S	CRISPR/Cas9 (Gene replacements and insertions) Should I include this here?	Glyphosate	Rice protoplast	(Li et al., 2016)
	T169I A170V P173S	Prime editing	Glyphosate	*Japonica rice*	(Li et al., 2020b)
	Triple substitution (T102I + A103V + P106S)	Screening naturally evolved variants via high-resolution melting analysis (HRMA)	Glyphosate	*Amaranthus hybridus*	(Perotti et al., 2019)
	G172A T173I P177S	Overexpression of resistant variants	Glyphosate	Japonica rice (*Nipponbare*)	(Achary et al., 2020)
Tubulin (*TubA2*)	M268T R243 M/K	rBE14 *Expression of* mutant α-tubulin gene	Dinitroanilines (Pendimethalin, trifluralin) Dinitroanilines (Trifluralin)	*Oryza sativa* *Lolium rigidum* (annual ryegrass)	(Liu et al., 2021) (Chu et al., 2018)
*OsPDS*	R304S R300S	Homologous recombination (HR)-mediated gene targeting (GT)/	Fluridone or Norflurazon	*Oryza sativa*	(Endo et al., 2021)
Glutamine synthetase (GS)	R295K R295K	DNA shuffling Transgenic overexpression	Glufosinate Glufosinate	*Oryza sativa* *Arabidopsis*	(Tian et al., 2015)
	A63V+P64S S59G+T60A	BEMGE	Gufosinate (PPT)	*Oryza sativa*	(Ren et al., 2023)
	–	Overexpression of OsGS1 or OsGS2 Co-overexpression of *OsGS1;1/OsGS2*	Gufosinate (PPT) limited tolerance to Gufosinate (PPT)	*Oryza sativa*	(James et al., 2018)
	S59G	Expression of naturally evolved variant	Gufosinate	Malaysian *E. indica*	(Zhang et al., 2022)

To establish continuous evolution of target genes in plant cells, TRACE has been employed in plant systems (**Figure 3B**). Remarkably high C:T editing efficiency was achieved in transient assays conducted in *Nicotiana benthamiana* (Butt et al., 2022). Acetolactate synthase (ALS), which is responsible for initiating the biosynthesis of the branched-chain amino acids such as valine, leucine, and isoleucine, serves as the primary target for at least five structurally distinct classes of herbicides (Shimizu et al., 2011). Point mutations were produced via targeting *OsALS* in rice by utilizing a chimeric fusion of T7-RNA polymerase and CBE guided by the specificity of the T7 promoter. By employing a T7 promoter-driven targeting the *OsALS* sequence, which was stably integrated into the rice genome, C:T and G:A transitions were generated. Subsequent application of bispyribac sodium (BS) as the selection pressure allowed herbicide-responsive residues within the *OsALS* sequence to be identified (Butt et al., 2022).

ALS has also been targeted with many SDE tools such as the Base Editing-Mediated Gene Evolution tool (BEMGE), which was constructed by combining nCas9, cytosine-adenine base editors, and an sgRNA library covering the full length of the *OsALS* coding region. Using this tool, base editors were guided by an sgRNA pool, resulting in a higher editing rate than when using samples transfected with a single gRNA. Additionally, four types of amino acid substitutions (R190H, P171F, P171L, and P171S) were obtained upon targeting, producing varying levels of BS herbicide resistance in plants (Kuang et al., 2020). As highlighted in another study, the Target-AID tool, which combines nCas9 with AID-BE, allows for precise base substitutions (specifically G:A or C:T) instead of random mutagenesis. The system was guided by sgRNAs directed toward the NGG PAM sequences located in the *OsALS* coding sequence, resulting in multiple mutations associated with herbicide resistance to imazamox in rice (**Table 1**) (Kuang et al., 2020).

Based on the concept of engineering allelic diversity, dCas9-BE can target the gene encoding acetyl-coenzyme A carboxylase, which catalyzes the first step of fatty acid biosynthesis in plants (**Figure 3C**). *OsACC*, which is the target of a major group of commercial herbicides, comprises 35 exons and encodes a protein consisting of 2327 amino acid residues. Herbicide-resistant-variants of this gene are located within the carboxyltransferase (CT) domain of *OsACC* (Jang et al., 2013). To comprehensively investigate *OsACC*, a pool of 141 sgRNAs was designed and introduced into rice callus, targeting the 1653-bp coding sequence of the CT domain situated in the 34th exon of *OsACC*. This was achieved using CRISPR-mediated base editing tools (eABE, eBE3, and eCDA), facilitating the identification of additional homozygous mutants that confer herbicide resistance in rice (**Table 1**) (Liu et al., 2020).

Another example of targeting herbicide tolerance genes *in planta* is the utilization of the STCBE-2 system, which facilitates near-saturated mutagenesis of *EPSPS*, which encodes 5-enolpyruvylshikimate-3-phosphate synthase, the target of glyphosate (**Figure 3D**) (Zhang et al., 2023). The STCBE-2 system consists of a dual cytosine and adenine base editor fused to nCas9-NG. Through multiple rounds of base editing and selections, researchers successfully obtained a novel allele of *OsEPSPS* that conveyed glyphosate tolerance to rice plants. This allele exhibited specific nucleotide changes that modified the coding sequence of *OsEPSPS*, particularly at position 213, where Asp was replaced by Asn (D213N). This alteration made the protein less susceptible to the inhibitory effects of glyphosate. The *OsEPSPS-D213N* modified allele was located in the predicted glyphosate-binding domain and provided robust glyphosate tolerance in rice (**Table 1**) (Zhang et al., 2023).

Other recent examples showcasing the efficacy of directed evolution involve the utilization of multiple versions of the CRISPR-based editing tool, such as SpCas9-NGv1, nSpCas9-NGv1 fused with APOBEC1, nSpCas9-NGv1-APOBEC-UGI, nSpCas9-NGv1-AID and nSpCas9-NGv1-AID-UGI. The SpCas9-NGv1-AID tool enabled the introduction of C:T substitutions into the *OsNGN, ALS*, *EPSPS* and the drooping leaf (*DL*) genes (Endo et al., 2019). The fusion construct efficiently induced mutations with variable efficiencies within the rice and Arabidopsis genomes (Endo et al., 2019).

## 3 Noncoding DNA and *cis*-regulatory elements

Although most approaches to date have targeted coding sequences for SDE of proteins, emerging technologies are beginning to examine the mechanisms regulating gene expression. Indeed, the diversity observed in crop characteristics arises from alteration not only in protein-coding genes but also in non-coding DNA elements (ncDNA). Modification in coding genes have the potential to disrupt protein structure and function, whereas alterations in non-coding DNA, specifically in *cis*-regulatory regions, regulate gene expression levels, thereby influencing a wide range of plant traits.

**Figure 4:**
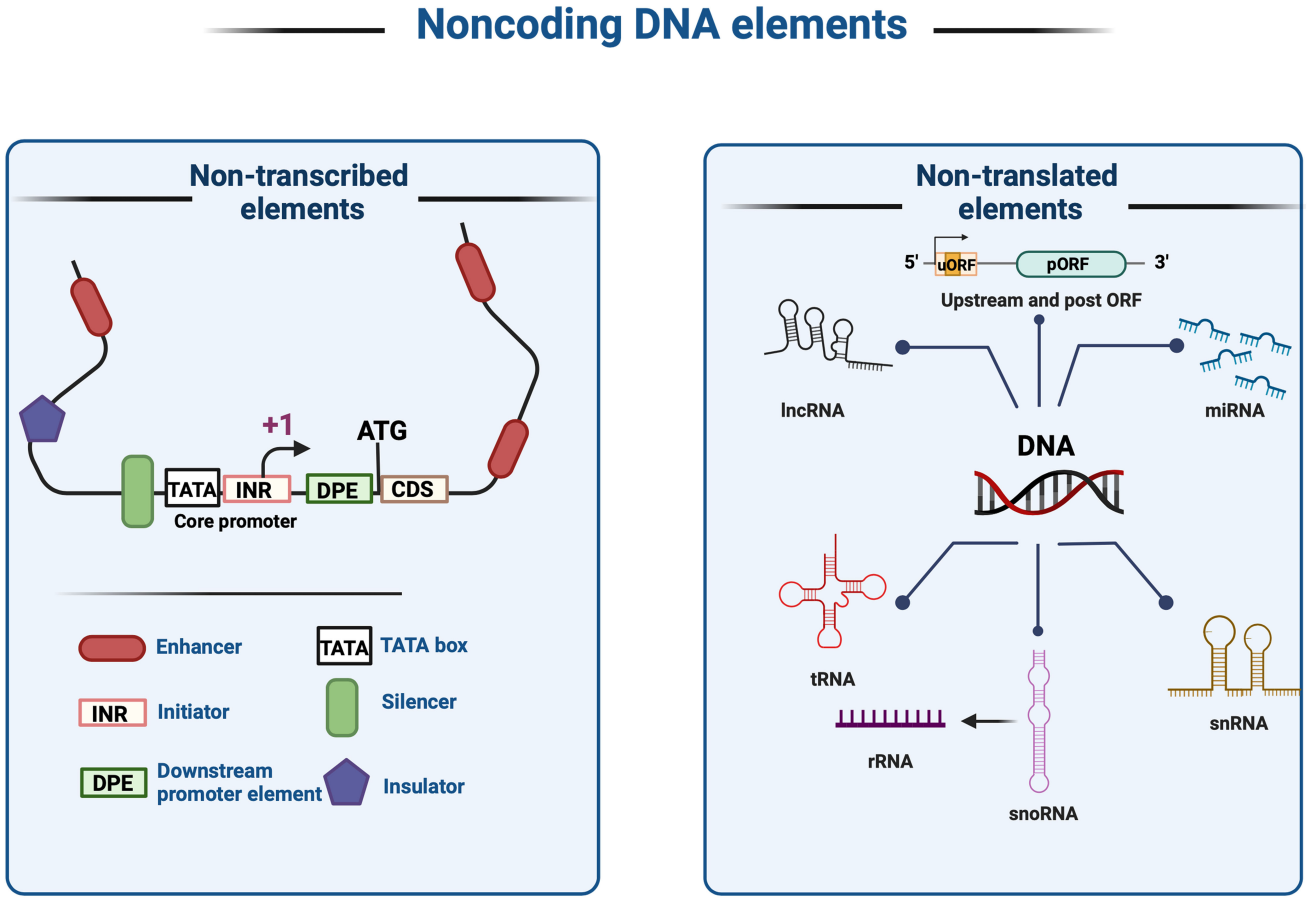
Noncoding DNA elements and cis-regulatory elements (CREs). Noncoding DNA elements can be classified into two broad categories: (1) Non-transcribed; elements that are not transcribed into RNA, including enhancers, initiators, downstream promoter elements, the TATA box, silencers, plus insulators that do not undergo transcription or translation; and (2) Non-translated; elements that are translationally silent noncoding elements, such as lncRNA, miRNA, tRNA, rRNA, and pre-mRNA.

Non-coding DNA elements are segments of DNA which does not encode proteins. ncDNA include two types of sequences: 1) non-transcribed, that remain un-transcribed but serve regulatory roles such as core promoters, enhancers, insulators, silencers, and response elements, which also known as *cis*-regulatory elements (CRE); or 2) non-translated, that transcribed into RNA, such as ribosomal RNAs (rRNAs), transfer RNAs (tRNAs), microRNAs (miRNAs), and long non-coding RNAs (lncRNAs) but not translated into proteins (**Figure 4**). CRE plays pivotal roles in regulating gene expression, modulating growth patterns, controlling spatial and temporal expression patterns, and regulating plant growth and development (Chekanova, 2015; Swinnen et al., 2016). In eukaryotic genomes, only 1% of the total DNA is responsible for encoding proteins (coding sequences), while the remaining 99% constitutes of non-coding DNA sequences. CREs are distinct DNA sequences recognized, for example, by transcription factors (TFs), which modulate transcription patterns. TFs bind to CREs, thereby activating or repressing the transcription of nearby genes. Regulatory elements ensure precise gene activation while preventing unwanted transcription. Coordinated actions of DNA-binding proteins and CRE interactions govern crucial cellular processes such as development and spatial patterning in planta (Jiang, 2015; Marand et al., 2023). Cells are tightly regulated by CREs to maintain proper gene expression and translation patterns. However, deciphering *cis*-regulatory roles and locations is challenging due to the vast diversity of regulatory sequences, epigenetic modifications, and environmental regulations (Schmitz et al., 2022; Biłas et al., 2016; Wittkopp and Kalay, 2011).

### 3.1 Computational tools and databases for predicting *cis*-regulatory elements in plants

**Figure 5:**
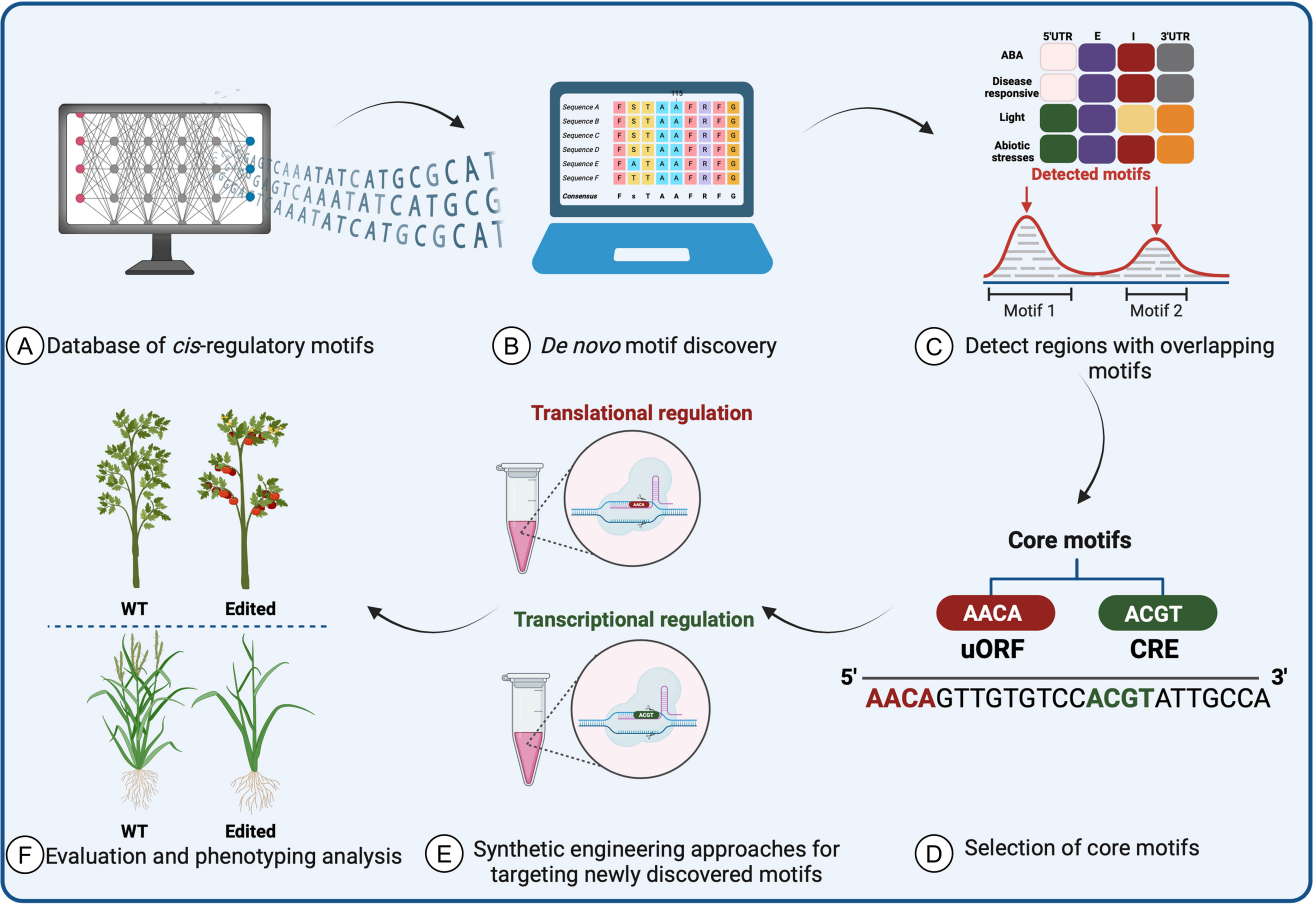
Prediction of *cis*-regulatory element motifs using computational approaches and databases in plants. **(A)** The required data are collected from validated databases and **(B)** converted into a matrix and structuring tool to form a baseline prediction tool. Following the discovery of motifs and *cis*-elements, **(C)** a search for homologs and overlapping motifs across species is performed. **(D)** After core motifs are identified and used in synthetic protein engineering methods to improve crops, **(E)** experimental validation and characterization are necessary to assess the accuracy of the data-driven machine-learning tools. **(F)** The data prediction tools will identify the most promising candidates for further testing as potential targets to improve crop traits.

To use SDE on CREs, researchers must first identify the CREs that regulate expression of the gene of interest in the relevant conditions and tissues. In recent years, there has been a notable focus on generating public databases and developing novel computational tools to study *cis*-regulatory roles and motifs *in planta*. These resources continually evolve, relying on systematic annotation with experimentally validated data to enable precise prediction of transcriptional networks. Integrating these predictions into plant applications is crucial both for conducting biologically significant analyses of regulatory sequence variation and for generating novel variants linked to desired phenotypes. This integration resolves issues such as annotation disparities, duplications, and standardized nomenclature, thereby enhancing the utility of these resources (**Figure 5**).

One of the earliest databases in plant research is PLACE (plant cis-acting regulatory DNA elements, which provides an accessible dataset of motifs within *cis*-acting regulatory DNA elements and their variants in vascular plants. These motifs are sourced from previous studies and are accompanied by concise descriptions. Additionally, variations of these motifs in different genes or plant species are documented in the PLACE database (Higo et al., 1998). Various studies have highlighted databases and prediction tools for predicting roles and locations of CREs. For example, the Database of Rice Transcription Factors (DRTF) is a valuable resource for studying TFs in *Oryza sativa*, providing comprehensive information on putative TFs, chromosomal localization, and sequence alignments. The DRTF dataset is supported by manual selection and computational predictions (Gao et al., 2006). AGRIS TRANSFAC is a database resource that provides valuable data on *Arabidopsis* promoter sequences, transcription factors, and their binding sites. It consists of two minor databases: AtTFDB, which focuses on *Arabidopsis* transcription factors, and AtcisDB, which is dedicated to *Arabidopsis cis*-regulatory elements, making the database more generically useful (Davuluri et al., 2003).

PlantCARE is a generic database identifying plant *cis*-acting regulatory elements, enhancers, and repressors, offering links to various databases such as EMBL, TRANSFAC, and MEDLINE (Lescot et al., 2002). An additional database generated for *Arabidopsis* is the Database of Arabidopsis Transcription Factors (DATF), which is specifically tailored for *Arabidopsis* transcription factors and encompasses 1827 genes spanning 56 families. Notably, DATF offers distinctive information and other features including 3D structure templates, expression information derived from ESTs, transcription factor binding sites, and nuclear localization signals (Guo et al., 2005). The Database of Poplar Transcription Factors (DPTF) is a collection of transcription factors compiled through a combination of computational predictions and manual selection in rice and *Arabidopsis* (Zhu et al., 2007). The Plant Transcription Factor Database (Plant-TFDB) provides information for TFs from various plant species, integrating high-quality non-redundant TF binding motifs and diverse regulatory elements and their interactions (Jin et al., 2017). Recently, Plant-TFDB, which has been developed to provide details concerning transcriptional regulation, expands its coverage across a diverse array of species for evolutionary annotation purposes, and highlights binding motifs along with TF functionality upon targeting (Tian et al., 2020). In the same study, they extended the development to generate the PlantRegMap tool, which focuses on functional regulatory mapping in plants. It assists in identification of transcriptional regulatory networks by predicting interactions between TFs and the target gene and by exploring TF binding sites as upstream regulators for the input gene (Tian et al., 2020). The recent PlantPAN 4.0 update is tailored to detect and analyze conserved non-coding sequences (CNSs) in plant promoters, providing valuable insights into the regulatory mechanisms underlying gene expression in a variety of plant species. Additionally, PlantPAN 4.0 can identify CNSs among related genes and explore different combinations and nucleotide variations of *cis*-regulatory elements, making it an essential tool for studying plant regulatory landscapes (Chow et al., 2024). As a proof of concept, seven bioinformatic models were employed to investigate *cis*-regulatory elements in soybean, *Arabidopsis*, maize, and rice (Zemlyanskaya et al., 2021; Ferebee and Buckler, 2023; Liu and Yan, 2019).

iCREPCP is a tool that employs deep learning techniques to identify CREs within plant core promoters, especially in maize and tobacco. By leveraging convolutional neural networks, iCREPCP accurately detects critical CREs that significantly contribute to promoter strength. This tool not only provides precise predictions of promoter strength but also identifies the position of each CRE with base-level resolution (Deng et al., 2023). A recent deep learning model, the Deep Learning Important Features (DeepLIFT) algorithm, was employed for accurately predicting regulatory effects based on genetic variation. DeepLIFT connects gene sequence data with mRNA copy numbers in various plant species, facilitating the identification of specific sequence features associated with gene expression (Peleke et al., 2024) (**Table 2**).

**Table 2:** Summary of computational approaches and databases for identifying plant transcription factors, core promoters, and UTRs.

Computational Tool/Database	Model	*cis*-regulatory elements	Links	References
Database of plant *cis*-acting regulatory DNA elements (**PLACE)**	Vascular plants	*cis*-regulatory regions, motifs, and homologs involved in plant regulation	http://www.dna.affrc.go.jp/htdocs/PLACE/	(Higo et al., 1998)
Database of Rice Transcription Factor (**DRTF**)	*Oryza sativa*	Provides comprehensive information on putative TFs, chromosomal localization, and sequence alignments of rice TFs	http://drtf.cbi.pku.edu.cn	(Gao et al., 2006)
Arabidopsis Gene Regulatory Information Server (**AGRIS**)	*Arabidopsis thaliana*	*cis*-regulatory elements, TFs, and putative binding sites	http://arabidopsis.med.ohio-state.edu	(Davuluri et al., 2003)
**PlantCARE**	Monocots, dicots and higher plants	Database of plant *cis*-acting regulatory elements, enhancers, and repressors within plant core promoters	http://sphinx.rug.ac.be:8080/PlantCARE/	(Lescot et al., 2002)
Database of Arabidopsis Transcription Factors (**DATF**)	*Arabidopsis thaliana*	TF binding sites, nuclear location signals, and 3D structure templates of ArTFs	http://datf.cbi.pku.edu.cn	(Guo et al., 2005)
The database of poplar transcription factors (**DPTF**)	*Populus trichocarpa, Oryza sativa and Arabidopsis thaliana*	TF prediction	http://dptf.cbi.pku.edu.cn	(Zhu et al., 2007)
The Plant Transcription Factor Database and prediction tool (**Plant TFDB**)	*Oryza rufipogon and O. nivara for O. sativa and green plants*	Identify and analyze plant TFs, regulatory elements, and their interactions	http://planttfdb.cbi.pku.edu.cn/	(Jin et al., 2017)
Plant Regulatory data and analysis platform (**PlantRegMap**)	Green plants	Charting plant transcriptional regulatory maps, prediction of regulatory interactions between TFs and input gene, and exploring of TF binding sites upstream regulators for the input gene	http://plantregmap.cbi.pku.edu.cn/	(Tian et al., 2020)
**PlantPAN 4.0**	*Arabidopsis thaliana, Oryza sativa, and Zea mays*	A tool for constructing transcriptional regulatory networks for diverse plant species	http://PlantPAN.itps.ncku.edu.tw/	(Chow et al., 2024)
**iCREPCP**	*Zea mays and tobacco*	A deep learning-based web server for single base-resolution *cis*-regulatory elements within plant core promoters	https://github.com/kaixuanDeng95/iCREPCP	(Deng et al., 2023)
Deep Learning Important Features (**DeepLIFT**)	*Arabidopsis thaliana, Sorghum bicolor, Solanum lycopersicum, and Zea mays*	UTR’s	https://github.com/JanZrimec/DeepExpression	(Peleke et al., 2024)

In contrast, there are few computational methods to predict the presence of enhancer sequences in plants. These databases, prediction tools, and computational models play crucial roles in advancing our understanding of plant transcriptional regulation and regulatory sequences roles, offering valuable insights into the intricate regulatory networks controlling gene expression and deepening our comprehension of plant evolution. Enhancers are crucial *cis*-regulatory elements capable of exerting control over genes from a distance, whether upstream, downstream, embedded within intronic sequences, or in close proximity to the coding sequence. As a proof of concept, predicted enhancers in intergenic regions were experimentally validated using reporter assays (Zhu et al., 2015) (**Table 3**). The self-transcribing active regulatory region sequencing (STARR-seq) method was employed to map functional enhancers and measure the enhancer activity of candidate sequences in rice. Approximately 9642 potential enhancers were predicted and identified through protoplast transfection of a genomic library derived from rice (Sun et al., 2019). For trait discovery, the STARR-seq analysis of the ATAC-seq (assay for transposase-accessible chromatin sequencing) library in maize unveiled enhancer activity in distal promoters linked with accessible chromatin regions and TF locations (Ricci et al., 2019). This signature-based enhancer prediction system utilized relative DNase-seq read enrichment in leaves compared to flowers to predict the tissue specificity of putative enhancers in *Arabidopsis*. For more precise and reproducible detection of enhancer activity, a variant of STARR-seq technology used for efficient identification of enhancers in transiently transformed *Nicotiana benthamiana* leaves. STARR-seq combined with site-saturation mutagenesis (SSM) to pinpoint functional regions within an enhancer, which recombined to create synthetic enhancers (Jores et al., 2020).

**Table 3:** Overview of Computational approaches and databases for identification of plant enhancers.

Computational Tool/Database	Model	*cis*-regulatory elements	Links	References
Global Quantitative Mapping of Enhancer Activity in Rice Genome by STARR-seq	*Oryza sativa L.* ssp *japonica*	Enhancers within the 5’UTR	Gene Expression Omnibus (GEO) as GEO: GSE121231	(Sun et al., 2019)
STARR-seq for genetic mapping	*Maize*	Transcriptional enhancer activity	https://github.com/schmitzlab/Widespread-Long-range-Cis-Regulatory-Elements-in-the-Maize-Genome/	(Ricci et al., 2019)
Signature-based enhancer prediction system	*Arabidopsis thaliana*	Enhancers in intergenic regions	BioProject/PRJNA252965	(Zhu et al., 2015)

### 3.2 Targeting *cis*-regulatory elements for crop improvement

Although CREs harbor a wealth of untapped potential variation for crop improvement, this aspect has remained largely unexplored until recently. Inducing precise mutations or producing a library of variants of these elements and motifs can lead to altered gene expression and induce phenotypic diversity within crop species. Understanding the role of CREs is crucial for unraveling the molecular mechanisms underlying their expression and function. These elements provide insights into the complex regulatory networks involved in producing valuable crop traits.

By manipulating CREs, researchers can fine-tune gene expression and modify plant traits, leading to alterations in plant morphology and overall plant architecture. For example, targeting the conserved *cis*-regulatory elements in the *SIKLUH* promoter using CRISPR/Cas9 system significantly reduced the proportion of small tomatoes and enhanced the weight of all fruits along the inflorescence (Li et al., 2022). The Waxy (Wx) gene encodes a granule bound NDP-glucosestarch glucosyltransferase in rice. Varied activities of natural Wx alleles regulate different amylose contents (AC), gel consistency (GC) and pasting viscosity of grain starches thus ultimately influencing the grain appearance and cooking/eating quality. Rice grains with higher ACs and lower GC values have poor eating quality, while those with moderate ACs and higher GC values give better taste for most consumers (Zeng et al., 2020). Disrupting the region near the TATA-box in the core promoter region of the *Wx* allele led to a moderate decrease in the amylose content in rice (Huang et al., 2020; Butardo et al., 2017). Editing of the *Wx* promoter to disrupt the A-box, CAAT-box, and 5′ UTR intronic splicing site (5′ UISS) produced *Wx* variants with variable amylose content (Zeng et al., 2020). Editing CREs, such as the RY-element and 2S seed protein motif of the fatty acid desaturase 2 promoter (FAD2), in peanut seeds manipulated its expression and improved the fatty acid profile by increasing oleic acid levels (Neelakandan et al., 2022).

### 3.3 Engineering promoters for crop improvement

The core promoter region varies among species and genes and is comprised of multiple elements. In eukaryotes, the TATA-box is crucial for minimal promoter activity. Additionally, elements like the initiator (Inr) sequence, transcription factor binding sites, downstream promoter element (DPE), and Y-Patch are distributed in diverse combinations, playing essential roles in facilitating efficient transcription factor binding and supporting the regulation of gene expression.

**Figure 6:**
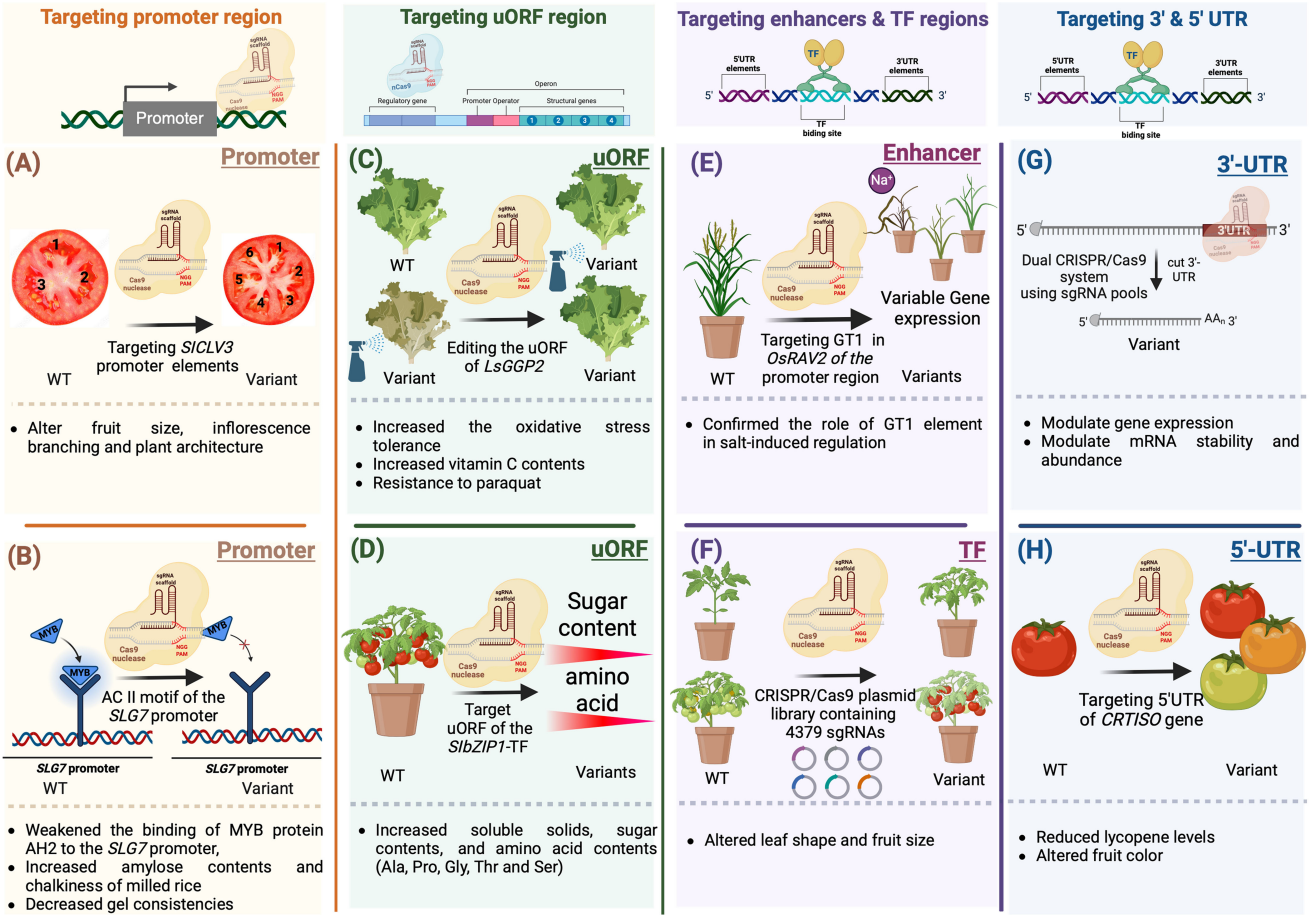
Gene editing of cis-regulatory elements and motifs for crop improvement. **(A)** CRISPR/Cas9-induced mutagenesis can introduce novel quantitative and qualitative variations for a variety of desirable traits, leading to observable differences in fruit characteristics **(B)** Prediction of cis-regulatory elements in the SLG7 promoter using Plant-CARE led to the generation of novel beneficial alleles for enhancing rice appearance. The AC II element-containing region of the SLG7 promoter was targeted via CRISPR/Cas9 gene editing This weakened the accessibility of MYB protein AH2 to the SLG7 promoter, resulting in increased amylose content, decreased gel consistency, and improved chalkiness of milled rice. **(C)** Induced mutations in the SC-uORF of the tomato transcription factor gene SlbZIP1 by the CRISPR/Cas9 system led to increased sugar and amino acid contents in tomato fruits. **(D)** A CRISPR/Cas9 system guided by dual gRNAs was employed to introduce targeted mutations in the uORF regions of SlbZIP1, which encodes a protein responsible for sucrose-induced repression of translation in tomatoes. Targeting the SC-uORF led to significant alterations in sugar and amino acid concentrations in tomato fruits, with amino acid levels increasing by up to 132% compared to WT fruits. **(E)** The GT-1 element plays a crucial role in facilitating adaptive salt responses through the OsRAV2 promoter. This was confirmed through *in situ* validation in plants with targeted mutations generated using the CRISPR/Cas9 system. This approach revealed the 65-bp region responsible for salt tolerance within the OsRAV2 promoter. **(F)** CRISPR/Cas9 plasmid libraries were utilized to target transcription factors in the tomato genome. Mutant populations were obtained by employing a large-scale pooled library of Agrobacterium plasmids for genetic transformation. Phenotypic changes observed in field-grown plants included alterations in leaf shape or number, seedling yellowing, modifications in fruit morphology or size, and abnormal development of floral organs **(G)** Recombinant Cas9 and *in vitro* transcribed gRNA can be used to target 3′-UTRs. This high-throughput approach identified regions that regulate mRNA abundance and mRNA stability. **(H)** CRISPR/Cas9 system was utilized to modify the 5’ UTR of CRTISO. This resulted in reduced levels of lycopene, a pigment responsible for the red color in tomatoes.

Modifying CREs can selectively eliminate specific motifs, resulting in manipulating transcript abundance, controlling expression patterns, and generating a series of phenotypic variants (**Figure 6**). For example, regulating meristem maintenance and determinacy in tomato was achieved by inducing targeted modifications in the promoter of *SlCLV3* (Rodriguez-Leal et al., 2017) (**Figure 6A**). Moreover, CRISPR/Cas9-induced mutagenesis can introduce novel quantitative variation for diverse traits, leading to observable differences in fruit size and inflorescence branching (Rodriguez-Leal et al., 2017). In a similar way, the prediction of *cis*-regulatory elements in the *SLG7* promoter using Plant-CARE led to the generation of novel beneficial alleles for enhancing rice appearance quality. Targeting the AC II element-containing region of the *SLG7* promoter via CRISPR/Cas9 gene editing weakened the accessibility of MYB protein AH2 to the *SLG7* promoter, resulting in increased amylose content, decreased gel consistency, and improved chalkiness of milled rice (**Figure 6B**) (Tan et al., 2023).

Similarly, mutagenesis of the effector-binding element (EBE) within the promoter of *SUGAR WILL EVENTUALLY BE EXPORTED TRANSPORTER* (*OsSWEET14*) using CRISPR/Cas9 conferred resistance to bacterial blight (Duy et al., 2021). Recent research used CRISPR/Cas12a mutagenesis to enhance resistance to blight bacteria. Rice leaf blight is caused by the bacterium *Xanthomonas oryzae pv. oryzae* (*Xoo*). The upregulated by transcription activator-like 1 (UPT) effector box in the promoter region of the rice *Xa13* gene plays a key role in *Xoo* pathogenicity (Yu et al., 2021). Specifically, mutating a critical bacterial protein-binding site within the UPT box of the *Xa13* promoter effectively abolishes bacterial expression, offering a promising CRISPR/Cas12a mutagenesis strategy to enhance rice resistance against bacterial pathogens (Yu et al., 2021).

The *TaVRN1-A1* promoter contains several regulatory sites namely, a VRN box, CArG box, and putative AG hybrid box associated with wheat vernalization. A tool termed A3A-PBE, comprising an enhanced human APOBEC3A-CBE fused to nCas9 (D10A), was developed to target *cis*-elements of the *TaVRN1-A1* promoter to produce variants with different vernalization requirements. A3A-PBE edited all cytidines in positions of the protospacer corresponding to the VRN-box site, thereby disrupting the binding site of bZIP TFs. Variants incorporating these modifications were generated in wheat, rice, and potato (Zong et al., 2018). Several studies have examined the pleiotropic rice gene *IDEAL PLANT ARCHITECTURE 1* (*IPA1*), which encodes the transcription factor *OsSPL14* and regulates various phenotypic traits such as tiller numbers, grain production, and panicle size. In this study, a tiling deletion-based CRISPR–Cas9 screen identified a 54-base pair *cis*-regulatory region in IPA1 that is when deleted, leads to increased grain yield per plant. Further studies revealed that the 54-bp deleted fragment serves as a target site for the transcription factor An-1, which represses IPA1 expression in panicles and roots (Song et al., 2022).

### 3.4 Engineering upstream open reading frames for crop improvement

Gene regulation extends beyond transcription to include posttranscriptional mechanisms that fine-tune gene expression. Elements operating at the translational level play a significant role in modulating the translation of downstream primary open reading frames (pORFs). The upstream open reading frame (uORF) is located upstream the coding gene and is not usually categorized as a non-coding DNA regulatory element. uORFs are recognized for their ability to influence translation, either positively or negatively, and sometimes initiate nonsense-mediated mRNA decay. In plants, approximately 30-40% of genes contain uORFs (von Arnim et al., 2014). In recent years, considerable attention has been given to studying the physiological roles of plant-specific uORFs, which have been extensively reported (van der Horst et al., 2020). Modifying uORFs through genetic editing tools can lead to the regulation of translation. This can be achieved either by introducing new uORFs or by extending the original uORFs through targeted modifications of their stop codons. For example, altering the uORFs in *OsDLT, OsTB1, OSBR1*, and *OsTCB19* via base editing and prime editing allowed control of gene expression and generation of a variant series exhibiting many phenotypical phenotypes in rice (Xue et al., 2023). Additionally, utilizing the CRISPR/Cas9 system for manipulating uORFs via genome editing allows fine adjustments in mRNA translation, thereby influencing protein concentrations. For example, uORFGDP-l-galactose phosphorylase (*LsGGP2*) was targeted, resulting in an 80-140% increase in ascorbate (vitamin C) content in lettuce (*Lactuca sativa*) leaves, as well as enhanced tolerance to oxidative stress (**Figure 6C**) (Zhang et al., 2018). In a recent study, the CRISPR/Cas9 system guided by dual gRNAs was employed to introduce targeted mutations in the uORF regions of *SlbZIP1*, which is responsible for sucrose-induced translation repression (SIRT) in tomatoes (**Figure 6D**). Targeting the SC-uORF led to significant alterations in sugar and amino acid concentrations in tomato fruits, with amino acid levels increasing up to 132% compared to WT plants. These induced mutations influenced the transcription levels of *SlbZIP1* and related genes involved in sugar and amino acid biosynthesis, resulting in varying mRNA levels in mutant tomato lines. This highlights how precise modifications of regulatory elements can manipulate the expression of key transcription factors, profoundly impacting vital fruit quality traits (Nguyen et al., 2023).

### 3.5 Engineering enhancers, transcription factors, 5′UTRs and 3′UTRs for crop improvement

Genetic variations in a transcription factor can influence TF binding, abundance, and activity. In a recent study, TFs linked to male sterility in maize were targeted. CRISPR/Cas9-mediated gene mutagenesis was employed to identify specific TFs regulating male sterility through genetic manipulation (Jiang et al., 2021). The GT-1 element in the *OsRAV2* promoter plays a crucial role in facilitating adaptive salt responses. This was confirmed through *in situ* validation with targeted mutations generated using the CRISPR/Cas9 system in plants. Initially, regulatory elements in the *OsRAV2* promoter were identified using databases and computational tools such as PLACE and PlantCare (**Figure 6E**) (Lescot et al., 2002; Higo et al., 1998). Subsequently, *Agrobacterium*-mediated transformation was employed to induce site-directed mutagenesis in the promoter region of *RAV2*, specifically targeting the GT-1 element in rice. This approach ultimately revealed the 65-bp region responsible for salt tolerance within the *OsRAV2* promoter (Duan et al., 2016). For genome-wide analysis, use of a CRISPR/Cas9-sgRNA library identified 990 transcription factors in the tomato genome and synthesized 4379 plasmid-sgRNAs for targeted modification. This library enabled the creation of variations in multiple transcription factors using the CRISPR/Cas9-*Agrobacterium* transformation method. The efficacy of the CRISPR/Cas9-TF mutant library was validated through the observation of diverse phenotypic changes, such as modified leaf shape or number, seedling yellowing, alterations in fruit morphology or size, and abnormal floral organ development (**Figure 6F**) (Bi et al., 2023).

Regulatory elements within the 5’ and 3’ untranslated regions (UTRs) of genes are known to play crucial roles in controlling gene expression and posttranscriptional processes (Li et al., 2015). A recent study utilized a dual-CRISPR/Cas9 system to target modifications of regulatory elements within 3’ UTRs (**Figure 6G**). This involved the use of a gRNA library for high-throughput localization of these elements, therefore targeting the full-length 3’ UTRs. A high-throughput approach was then employed to identify regions regulating mRNA abundance. Individual gRNAs were subsequently used to screen the impact of targeting these regions on transcription and mRNA stability (Zhao et al., 2017). The CRISPR/Cas9 system was also utilized to modify the 5’ UTR of *CRTISO*, which encodes carotenoid isomerase in tomato. By introducing genetic modifications in the 5’ UTR region, researchers were able to manipulate the expression level of *CRTISO*. This resulted in reduced levels of lycopene, a pigment responsible for the red color in tomatoes (**Figure 6H**) (Lakshmi Jayaraj et al., 2021).

These studies emphasize the importance of utilizing SDE through genome engineering tools to investigate, understand, and characterize the functional significance of regulatory elements. Direct manipulation of these regions allows researchers to uncover the intricate mechanisms governing gene regulation and potentially devise novel strategies for crop enhancement and trait modification.

## 4 Conclusions

Genetic variation provides fuel for plant breeding and, by harnessing synthetic directed evolution (SDE), diverse alleles can be generated for crop improvement. SDE tools not only modify the coding regions but also can be used to engineer non-coding regions to develop novel traits in crop plants. In this review, we have summarized the methodologies and strategies for protein engineering via SDE. These SDE tools were initially developed in bacteria but have been adapted to eukaryotic systems and are now used for plant trait engineering. Although SDE efforts have essentially focused on protein engineering and on recovering variants associated with desired traits, this represents only a small fraction of the possible variation that could be engineered under selective pressure. Other noncoding genetic elements that positively or negatively modulate transcriptional activity, including promoter sequences, uORF, Transcription Factors, 5´-UTR, and 3´-UTR, and *cis*-elements, could also be used for SDE. There is a huge potential to target and engineer the non-coding regions and *cis*-regulatory elements for trait improvement in plants, enabling us to tackle the current challenges of climate changes and global food production.
